# Beyond access to medicines: Eliciting high–income country support for a new global health research and development paradigm

**DOI:** 10.7189/jogh.03.020303

**Published:** 2013-12

**Authors:** Sadie Regmi, Benjamin Skov Kaas–Hansen, Johanne Helene Iversen

**Affiliations:** 1 Institute for Science, Ethics and Innovation, University of Manchester, United Kingdom; 2 Faculty of Health Sciences, Aarhus University, Denmark; 3 Faculty of Medicine and Dentistry, University of Bergen, Norway

In recent decades, debates surrounding access to medicines have moved on to discussions on new ways of conducting research and development (R&D) to ensure equitable access from the outset. The market failure in the current R&D system and the need for new models has been apparent for decades [1,2]. A new framework for research and development to address health gaps primarily affecting low– and middle–income countries (LMICs) is currently one of the most contentious issues being debated at the World Health Organization (WHO). While WHO member states agree that urgent action is needed, deciding upon models, implementation mechanisms and funding commitments has proven difficult [3].

The report of the Consultative Expert Working Group on Research and Development: Financing and Coordination (CEWG), published in 2012, marks the most recent milestone in the search of a new R&D paradigm (Box 1) [7]. The CEWG was set the task of finding a solution to lack of funding for diseases that are not catered for by today’s market forces, and they did so laudably, analysing various proposals in great depth and impressively dealing with conflicts of interest. Requiring nations to make final commitments is inevitably a difficult ask in today’s economic climate. Even so, framing the recommendations as purely a set of models and mechanisms to close the gaps in accessible health care technology for the poor and failing to highlight the benefits for high–income countries (HICs), may have undermined crucial support for a new R&D paradigm. We aim to point out the benefits HICs will accrue from adopting a new R&D framework. These benefits fall into three categories: increased investment by middle–income countries (MICs) into R&D so that HICs get increased returns on current investment; a more sustainable and efficient funding source for R&D; and direct benefits through the products of R&D into new antibiotics and vector borne diseases.

Box 1 **The Consultative Expert Working Group on Research and Development: Financing and Coordination (CEWG)**

**WHAT**

The CEWG was established by the 63^rd^ World Health Assembly in 2010 and consisted of professionals with a wide variety of expertise. The final report was published in April 2012 and included a set of key recommendations to address the gaps in health R&D [1,4].

**WHY**

Securing access to affordable health technologies in low– and middle income countries continues to be among the greatest challenges in global health. Underpinning the challenge is a systematic market failure in health R&D which leads to an underproduction of public goods. Incentives such as intellectual property (IP) rights have traditionally been used to address this underproduction. However, the IP model, which incentivises private industry to invest in health R&D provided they get a monopoly on the end product, has failed to provide incentives for the development of health technologies addressing diseases that primarily affect the poor.

**KEY RECOMMENDATIONS**

**Approaches to R&D:**

– Open knowledge innovation, equitable licensing and patent pools should be embraced

**Funding mechanisms:**

– Countries should commit to spend 0.01% of GDP on government funded R&D to meet the health needs of the poor

**Pooling resources:**

– 20–50% of the funds raised should be channelled through a pooled mechanism

**Coordination:**

– A global health observatory under the auspices of WHO should be established [5,6]

**Implementation:**

– A binding global instrument for health R&D and innovation should be implemented

– Formal negotiations on an international convention should be initiated.

**CHALLENGES**

All member states agree that the market failure in health R&D is a pressing global health challenge. However, member states, in particular high–income countries, have been reluctant to support concrete, binding commitments. The most contentious issues have been the financing commitment of 0.01% of GDP and the suggested implementation through a binding convention.

## NO MORE FREE–RIDING – SHARED RESPONSIBILITY OF ALL NATIONS

One of the arguments for the globalisation of intellectual property (IP) rights through the World Trade Organizations (WTO) agreement on Trade–Related Aspects of Intellectual Property Rights (TRIPS) in 1994 was that LMICs were able to “free–ride” on the research conducted in HICs [8]. The proponents of this argument held the view that LMICs, whilst benefiting from the products of the translation of research, failed to contribute to it. By setting global standards for IP protection, this “free–riding” has been addressed to a degree as LMICs (particularly MICs) now largely abide by global IP protection standards, many due to the threat of economic sanctions [1]. However, this has come at a cost: access to essential medicines for the world’s poor has been jeopardised [2]. The impact of the globalisation of IP rights has been the subject of much debate. Many have questioned the wisdom of requiring countries that lack even basic health infrastructure to adhere to global standards in intellectual property [1,9]. Such debates have undoubtedly influenced the quest for new mechanisms to incentivise global health innovation [10].

Currently, the US is the world’s largest contributor to R&D addressing diseases that primarily afflict the poor. It is the only nation that meets the 0.01% of GDP minimum expenditure advocated by the CEWG [11]. Most MICs, despite having made significant gains in GDP, have arguably not contributed their fair share to R&D into diseases that primarily afflict their populations [11]. A global commitment to increase public R&D funding would enable pooling of funds from all participating nations, thus addressing the so–called free–rider problem. Countries which already contribute vast amounts would see a greater return on investment. Moreover, an equitable and sustainable model would be created, replacing a framework based on charitable motives of HICs with one based on shared responsibility of all nations. It would also ensure that all contributing member states take ownership of investments and outcomes, a necessity for sustainability.

Knowledge created by research is a global public good. But for knowledge to be a true global public good, access has to be non–rivalrous and non–excludable: that is, without restrictions and freely available to all [12]. For this to happen, states have to take a greater part in upfront financing of research generated knowledge; legislative measures must be put in place to ensure that such knowledge is in fact accessible to all [13]. By making knowledge derived from research more widely available, the global community has the potential to minimise – and perhaps in time eliminate – duplicative research [14]. Duplicative research slows the knowledge generating process and increases expenses associated with research [15]. According to Subra Suresh, Director of the United States National Science Foundation, “More nations recognize that innovation, driven by science and engineering (S&E), is the fuel for economic growth, prosperity, and social well-being.” [16]. As more actors from across the world increase contributions to the R&D landscape, minimising duplication of research will be ever more vital.

With the financial crisis sweeping the world, limited resources are available for R&D. These resources have to be coordinated effectively and prioritised to meet the greatest health challenges posed by the global burden of disease [17]. The CEWG suggested a convention under the auspices of WHO to ensure sustainable global governance for R&D, whereby member states, through a coordinating organ will be given the responsibility of prioritising and allocating funding.

HICs already have significant experience in advanced research, innovation, and technology transfer. They could benefit from a model with pooled funds, because the funds are likely to flow into existing research facilities, which could bring a boost for existing research projects and incentivise new ones.

## MOVING TOWARDS SUSTAINABLE HEALTH RESEARCH FUNDING

Pharmaceuticals comprise a significant proportion of health expenditure; the pharmaceutical bill across the OECD countries was estimated to have reached more than US$ 700 billion, accounting for around 19% of health spending. In an attempt to cut overall health costs, many European countries made efforts to control pharmaceutical expenditure before the economic recession through a mix of price and volume controls directed at physicians and pharmacies, as well as policies targeting specific products [18].In this context, it is vital that the process of pharmaceutical R&D is made as efficient as possible.

The traditional models of incentivising innovation are not delivering, even for HICs [19]. The current IP system does not incentivise innovation on the basis of need, but possible profit. Lifestyle drugs and me–too drugs flourish whereas much–needed treatments receive little funding [20]. Many companies secretly pursue similar lines of research hoping to be the first to get a product patent. Pharmaceutical companies, which are primarily responsible to their shareholders, cannot be solely blamed. Structural mechanisms that do not reward needs–driven innovation must be rectified. Moving to a system that encourages openness by its very nature would reduce inefficiencies and cut costs [14,21,22]. The problem, then, would lie with incentivising translation of basic research to commercial products [10]. HICs, therefore, have an interest in ensuring that a significant proportion of the pooled funds end up as prize funds accessible to both private and public institutions. If such a model, based on openness, proves successful and minimises inefficiencies, there is potential for expanding the model.

## IN THE SAME BOAT – HOW GLOBALISATION TRANSFORMS DISEASE PATTERNS

Globalisation plays an increasingly important role in human health and security [23]. With modern transportation technologies, migration and travelling have become vastly easier than a decade or two ago. People move easily between continents and, consequently, so do diseases. Indeed, many communicable diseases can now spread across countries and continents within their incubation periods, exemplified by the severe adult respiratory syndrome outbreak in 2002 and 2003, when only a concerted international effort prevented the spread from reaching pandemic proportions [24]. It is not just infectious diseases that easily traverse borders; it seems sedentary lifestyles and habits spread almost as easily. The rise of non–communicable diseases is a major global public health threat that must be addressed through shared global action, both when it comes to prevention and treatment [17].

**Figure:**
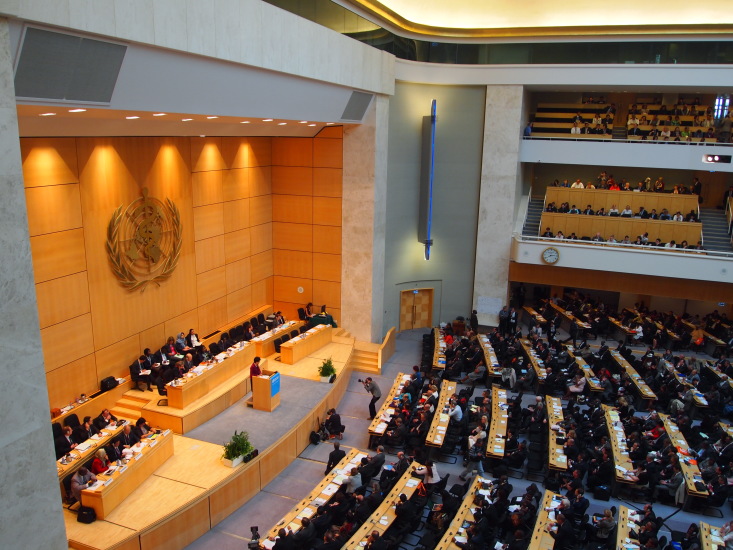
Photo: Courtesy of Sadie Regmi, personal collection

Disease patterns in the world have changed significantly, not just because of the movement of people, lifestyles and norms, but also the movement of disease–carrying vectors due to a changing climate. For instance, West Nile Virus is spreading across the US at an increasing pace, having affected thousands of Americans in just a decade [25]. Similarly, the incidence rates of dengue fever in the US have increased greatly, and two types of mosquitoes capable of transmitting the dengue virus can now be found in 28 states. With further changes to the climate and the following rise in average temperature, this trend is expected to continue [26].

The spread of drug resistant microbes is another major global concern. There is a large discrepancy between the burden of infectious due to multidrug-resistant bacteria and investments into the development of new antibiotics. According to the Global Risks 2013 report issued by World Economic Forum the global risk of antimicrobial resistance is linked to the “failure of the international Intellectual Property (IP) regime” [27].

These public health concerns are not country-specific and dealing with them will require concerted global efforts. Indeed, with increased globalization, it is perhaps time we moved beyond the notion of viewing some diseases and challenges as being *tropical*. Recent history suggests that diseases which today primarily afflict populations in LMICs can evolve into true global public health threats imposing significant financial strain on health care systems in high–income countries as well.

## CONCLUSIONS

The world is in transition: countries which are powerful today will not necessarily remain as powerful tomorrow, diseases are moving beyond geopolitical borders, and the threat of common antimicrobials becoming ineffective is a pressing public health issue for all. Today’s R&D system is clearly not optimal: drug pipelines are not aligned with global health needs, prices for end–products are becoming ever more unaffordable, and inefficiency and declining rates of true innovations are becoming hallmarks of the R&D process. These patterns have emerged over the last few decades and despite many perceptive analyses of the situation, action towards repairing the situation remains wanting.

The new R&D framework suggested by the CEWG addresses some of the crucial problems in health R&D as it encompasses a number of models that could improve the existing system. Unfortunately, most HICs have so far expressed little will to truly explore the possibility of a global shift in norms. We believe one factor contributing towards such a response is the failure to highlight the potential benefits for these countries.

There are still several unanswered questions concerning how such a new R&D framework could work: how much would each country have to contribute to make the fund large enough, and how much would need to be pooled? How many countries would need to participate? How will the coordinating organ be organised and whom should it consist of?

Wider political interest in global health has meant that the health community currently has the opportunity to debate health R&D within a wider political arena; the CEWG’s work is a solid platform on which to base these debates. WHO member states have started to identify promising demonstration projects that address the health R&D gaps through incorporation of open knowledge innovation mechanisms. This is a welcome start, but questions surrounding sustainable financing and ability or willingness to scale up such projects within a new normative framework still remain unanswered. A new R&D framework will not address every health challenge we face, but it could be a first step towards creating a sustainable system for pharmaceutical R&D where nation states bear shared responsibility for global public health.
